# The Impact of the Time Interval Between Radiation and Hyperthermia on Clinical Outcome in Patients With Locally Advanced Cervical Cancer

**DOI:** 10.3389/fonc.2019.00412

**Published:** 2019-05-21

**Authors:** Hans Crezee, H. P. Kok, Arlene L. Oei, Nicolaas A. P. Franken, Lukas J. A. Stalpers

**Affiliations:** ^1^Department of Radiation Oncology, Amsterdam University Medical Centers, University of Amsterdam, Amsterdam, Netherlands; ^2^Laboratory of Experimental Oncology and Radiobiology, Amsterdam University Medical Centers, University of Amsterdam, Amsterdam, Netherlands; ^3^Center for Experimental Molecular Medicine, Amsterdam University Medical Centers, University of Amsterdam, Amsterdam, Netherlands

**Keywords:** radiotherapy, hyperthermia, time interval, DNA damage repair, reoxygenation

The benefit of hyperthermia combined with radiotherapy is well-acknowledged for patients with locally advanced cervical cancer (LACC) (1–3). However, recently a discussion evolved on the optimal time interval between radiotherapy and hyperthermia. Kroesen et al. (4) recently reported a retrospective analysis of factors influencing clinical results of treatment with radiotherapy and hyperthermia in a large cohort of locally advanced cervical cancer patients (LACC) at ErasmusMC in Rotterdam. They concluded that there is no detrimental effect of prolonged intervals on clinical outcome within a time frame of 4 h between radiotherapy and hyperthermia. Kroesen et al. thereby explicitly dismissed the findings of Van Leeuwen et al. (5) in a smaller cohort of LACC patients treated at the Academic Medical Center (AMC) of the University of Amsterdam. In that study longer time intervals and lower tumor temperatures were both found to have a highly negative effect on in-field tumor control (time interval: *p* = 0.021, in multivariable analysis *p* = 0.007) and overall survival (idem: *p* = 0.015, in multivariable analysis *p* = 0.012), where it is important to note that the median time intervals between radiotherapy and hyperthermia were ~60 and ~90 min for the short and long time interval subgroups of patients, respectively.

We feel that the conclusion of Kroesen et al. is presented with insufficient caution. The absence of an impact of time interval may be true in their cohort, but that does not mean that time interval never plays a role for LACC patients treated with radiotherapy and hyperthermia. We are inclined to attribute that difference in outcome to a different mix in working mechanisms and patient population in the ErasmusMC cohort and in the AMC cohort.

Multiple working mechanisms contribute to the effectiveness of hyperthermia, as was also nicely summarized by Kroesen et al. Relevant is that each of these mechanisms require a different optimal temperature range. For instance, inhibition of DNA repair is a very effective radiosensitizer, but requires at least 41°C (6–8), a significantly higher temperature than required for many other mechanisms, such as the reperfusion mechanism leading to sensitization through reoxygenation, which will occur at more moderate temperatures starting at 39°C (9, 10). Thus, the tumor temperature achieved should be sufficiently high for a significant contribution of inhibition of DNA repair to the overall hyperthermia effect, and the question is whether this level was achieved in the study of Kroesen et al. where the median temperature rise was 3.5°C, equivalent to a median tumor temperature below 40.5°C (4), in line with the median vaginal lumen temperature of 40.3°C reported for a similar large cohort of LACC patients from ErasmusMC (11), which partly overlaps the present ErasmusMC cohort. Van Leeuwen did not report the median vaginal lumen temperature, but instead a measure for the minimum temperature: T90 = 40.2°C, with T90 the temperature exceeded in 90% of the volume, equivalent to a median temperature close to 41°C. Kroesen et al. attribute the lack of impact of time interval on clinical results to either no contribution of inhibition of DNA repair to the hyperthermia treatment effect, or to a fairly time interval-independent contribution by assuming the hyperthermic inhibition of repair involves very slow DNA damage repair processes, up to 6 h. Much DNA damage is repaired by fast repair within an hour after radiotherapy, repair of the residual damage can indeed take more hours (6, 12, 13). Study of DNA damage repair kinetics in cervical cancer biopsies did suggest the majority of DNA damage is repaired within 2 h though (5). But even if hyperthermia had contributed in the cohort of Kroesen et al. by inhibiting very slow DNA repair processes taking up to 6 h, then differences in effectiveness of hyperthermia should have been visible when comparing the shortest (0.5–1 h) and longest (1.5–4 h) time interval subgroups shown in Figure 2 of (4), as even for a very slow repair of 6 h at least half the DNA damage should have been repaired in the longest time interval subgroup, vs. minimal repair for the shortest time interval subgroup. This suggests near absence of DNA repair inhibition is the most likely explanation for the lack of effect of time interval found in the ErasmusMC cohort.

Overgaard (14, 15) found for hyperthermia combined with radiotherapy in an *in vivo* murine model significant contributions of two clearly different working mechanisms: one fairly independent of the time interval, which probably reflects the dominant mechanisms also present in the LACC patients of Kroesen et al., and another mechanism only active when the time interval is shorter than 4 h, the latter is probably associated with inhibition of DNA damage repair, augmenting the effect of radiotherapy. The latter mechanism also showed a significant increase in thermal enhancement when the time interval was shortened from 4 to 1 h and even to 0.5 h, in agreement with the clinical results at AMC. This rapid increase of the thermal radiosensitization with shorter time intervals has been successfully used in a study using very low-dose hypofractionated weekly re-irradiation sessions (5 x 4 Gy) immediately following hyperthermia treatment for recurrent breast cancer patients (16). The temperature used by Overgaard was 42.5°C and it is clear that the contribution of inhibition of DNA damage repair will eventually drop to zero when the tumor temperature is gradually decreased to 41°C. A good hyperthermia effect is of course still possible without inhibition of DNA damage repair. Tumor hypoxia is a serious factor in treatment failure, particularly in LACC, and the reoxygenation effect of hyperthermia may overcome this tumor hypoxia and thereby significantly enhance the effect of radiation, as also noted by Dewhirst et al. (9). Direct cell kill of hypoxic tumor cells by hyperthermia will also contribute to enhancing the effect of radiation, an effect that also exhibits a clear dose-effect relationship (10). For this purpose a 4 h time interval is acceptable, but one should bear in mind that only part of the synergistic hyperthermia working mechanisms are utilized at these somewhat milder temperatures.

Our conclusion would be that inhibition of DNA damage repair appeared to be exploited less in the patients treated in the ErasmusMC cohort than in the AMC cohort. There is sufficient evidence to conclude that time interval does play a role in the application of radiotherapy and hyperthermia. Therefore, the conclusion of Kroesen et al. that prolonged time intervals between radiotherapy and hyperthermia are not detrimental to clinical outcome cannot be generalized.

## Author Contributions

HC drafting the article, critically revising the article. HK, AO, NF and LS critically revising the article.

### Conflict of Interest Statement

The authors declare that the research was conducted in the absence of any commercial or financial relationships that could be construed as a potential conflict of interest.
